# The association of telomere attrition with first-onset stroke in Southern Chinese: a case-control study and meta-analysis

**DOI:** 10.1038/s41598-018-20434-w

**Published:** 2018-02-02

**Authors:** Jing Li, Congrui Feng, Liang Li, Shujun Yang, Yu Chen, Rutai Hui, Mei Zhang, Weili Zhang

**Affiliations:** 1 Department of Neurology, The First People’s Hospital of Huainan, Huainan, 232007 Anhui province China; 2 Beijing Institute for Brain Disorders, Center for Brain Disorders Research, Capital Medical University, Beijing, 100069 China; 3 State Key Laboratory of Cardiovascular Disease, Fuwai Hospital, National Center for Cardiovascular Diseases, Chinese Academy of Medical Sciences and Peking Union Medical College, Beilishi Road 167, Xicheng District, Beijing, 100037 China; 4 Department of Surgical Intensive Care Unit, Fuwai Hospital, National Center for Cardiovascular Diseases, Chinese Academy of Medical Sciences and Peking Union Medical College, Beilishi Road 167, Xicheng District, Beijing, 100037 China

## Abstract

The relationship between telomere length and stroke was inconsistent mostly due to different pathogenesis of subtypes, environment and genetics. We aimed to assess whether leukocyte telomere contributes to stroke in Southern Chinese by investigating a case-control study comprising 543 cases (224 atherothrombotic stroke, 94 hemorrhagic stroke and 225 lacunar infraction) and 616 controls and replicated the investigation in an independent study comprising 773 cases and 875 controls with the same diagnostic criteria. Telomere was inversely correlated with increasing age in controls (correlation coefficient γ = −0.28, *P* < 0.001) and in cases with atherothrombotic stroke (γ = −0.17, *P* = 0.012). Individuals within the lowest tertile of telomere showed a higher risk for atherothrombotic stroke [odds ratio 2.33, 95% confidence (CI) 1.42–3.83; *P* = 0.003], whereas had a lower presence of lacunar infarction (OR 0.49, 95% CI 0.30–0.81; *P* = 0.007). Similar results were obtained in the second replication study. A further meta-analysis showed a 12% increased pooled risk of ischemic stroke (95% CI 1.04–1.18) in relation to shorter telomere, but this association was stronger in the retrospective studies and in Asians when stratified by study design and ethnicity. Our data provided the first evidence that in Southern Chinese stroke population, leukocyte telomere is independently associated with atherothrombotic stroke and lacunar infarction.

## Introduction

Stroke, prone to elderly, is one of major causes of mortality and disability worldwide and in China. Chronological age is an important predictor of cardiovascular disease including stroke, however, biologically cellular aging may be better at predicting risk of vascular diseases which particularly imply the accumulation of vascular damage over the lifetime, such as exposure to oxidative and inflammatory stress^1,2^. Although traditional risk factors such as smoking, drinking, dyslipidemia, diabetes, etc., explain most of stroke, there is wide difference in the onset of stroke and clinic features at an individual level, even in those persons with similar risk factor profiles. The inter-individual difference in biological aging is potential to affect the susceptibility to stroke.

Telomere, a specialized DNA-protein composite structure with repetitive nucleotides sequence ‘TTAGGG’ at the end of each chromosome, has a key role in preserving the stability and integrity of chromosome end^3^. Attrition rate of telomere represents the biological and functional status of a person more precisely than chronological age, and hence has been an appealing research target. Emerging evidence has clearly established that age-dependent telomere shortening of circulating leukocytes is correlated with atherosclerosis and cardiovascular diseases^4–7^, indicating that leukocyte telomere is a valuable biomarker to represent the biological aging of vascular system. In addition, studies by Wilson *et al*.^8^ and Zhang *et al*.^9^ showed that leukocyte telomere can serve as an appropriate indicator of telomere in vessel wall as well as in human atherosclerotic plaque tissues.

To date, studies have not drawn convincing conclusions with respect to the relationship of telomere length with stroke risk. These studies differed in study design, end-point definitions for stroke, follow-up years, and ethnicity of the population. Two nested case-control studies from USA have not found the positive association between leukocyte telomere and stroke^10,11^, whereas prospective studies from USA showed positive association between shorten telomere and increased risk of stroke, but not mortality in stroke^5,12,13^. Another study from European showed no relation between leukocyte telomere and stroke in elderly high-risk hypertensive patients^14^. A positive susceptibility to the risk of ischemic stroke and all-cause mortality related to telomere length has recently been reported in the Chinese population^9,15,16^. In China, the prevalence of stroke is markedly higher in the North (486 per 100,000 person-years) than in the South (136 per 100,000 person-years) because of different lifestyles, genetics and environments^17,18^. Telomere length has been observed a remarkable difference across the geographical areas of Europe^19^, however, pathogenesis underlying the geographical difference of stroke risk in China has not been investigated.

In this study, we aimed to further assess the relation between leukocyte telomere and stroke and subtypes in a Southern Chinese cohort using a case-control study design in Huainan city, Anhui province at southeast of China. A replication study was conducted in an additional independent case-control stroke cohort enrolled from November 2000 to November 2001 from 3 clinic centers located at Wuhan city and Chongqing city in the Southern China. The criteria of diagnosis, recruitment and collection of clinical characteristics were same in the two studies. In addition, we performed a meta-analysis and systematic review of previous prospective and retrospective studies.

## Results

### Characteristics of studied participants

**Figure 1:**
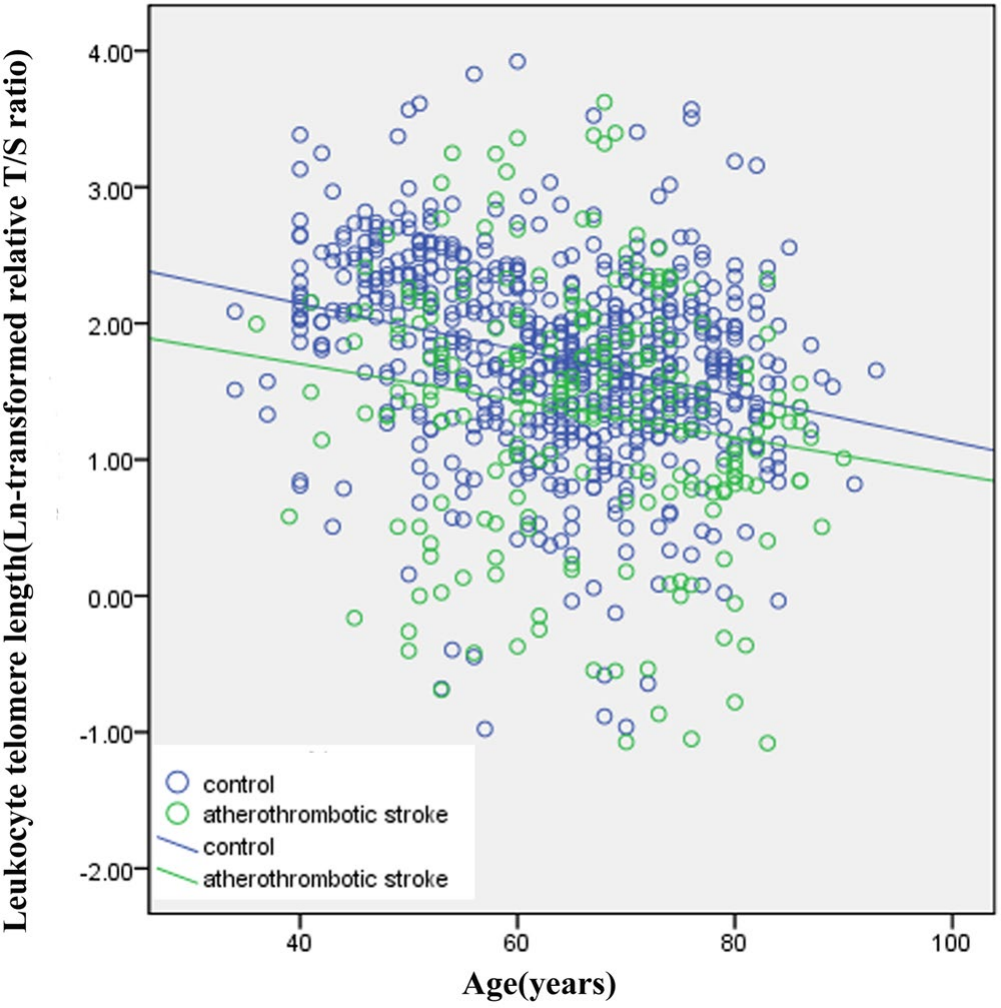
Telomere length as a function of age in control subjects and patients with atherothrombotic stroke Controls are shown as blue circles (n = 616) and atherothrombotic stroke cases as green circles (n = 224). Telomere length is expressed as the natural log (Ln) of relative telomere to single-copy gene (T/S) ratio. Slope of the line indicates the annual telomere-shortening rate in control subjects (coefficient correlation γ = −0.28, *P* < 0.001; blue line) and cases with atherothrombotic stroke (γ = −0.17, *P* = 0.012; green line), respectively.

In the first case-control stroke study, patients with stroke were more likely to have traditional risk factors, such as alcohol intake, smoking, hypertension, coronary heart disease, and higher blood pressure, dyslipidemia, fasting glucose, and serum uric acid (Table 1). There was significantly inverse correlation between leucocyte telomere and increasing age in control subjects (correlation coefficient γ = −0.28, *P* < 0.001) and in cases with atherothrombotic stroke (γ = 0.17, *P* = 0.012) (Fig. 1), but not in patients with intracerebral hemorrhage (γ = −0.06, *P* = 0.55) or lacunar stroke (γ = −0.12, *P* = 0.09). We observed that mean leukocyte telomere was remarkably lower in the atherothrombotic stroke patients (median 1.56, inter-quartile ranges 0.93–2.02) when compared with control subjects (median 1.76, inter-quartile range 1.34–2.23). But the telomere length ratio was markedly higher in lacunar stroke patients (median 1.93, inter-quartile range, 1.26–2.41).

**Table 1:** Clinical characteristics of the first case/control study in Southern Chinese population.

Characteristics	Control subjects (n = 616)	Stroke patients			
		Total cases (n = 543)	Hemorrhagic stroke (n = 94)	Atherothrombotic stroke (n = 224)	Lacunar infarction (n = 225)
Age, years	63.6 ± 11.9	65.9 ± 12.2**	63.5 ± 13.8	65.9 ± 11.6*	70.0 ± 11.9**
Male, n (%)	284 (46.1%)	336 (56.3%)**	54 (36.5%)	121 (54.0%)*	130 (57.8%)*
Body mass index, kg/m^2^	24.00 ± 2.28	23.90 ± 2.89	23.86 ± 2.91	24.18 ± 2.97	23.66 ± 2.77
Systolic BP, mmHg	136 ± 10	146 ± 23**	164 ± 28**	143 ± 19**	143 ± 22**
Diastolic BP, mmHg	83 ± 8.23	88 ± 14**	99 ± 18**	86 ± 11**	86 ± 12**
Glucose, mmol/L	5.41 ± 1.59	6.19 ± 2.52**	6.82 ± 3.38**	6.31 ± 2.17**	5.81 ± 2.37*
Total cholesterol, mmol/L	5.16 ± 0.98	4.64 ± 1.99**	4.91 ± 1.36*	4.68 ± 2.64**	4.49 ± 1.35**
Triglycerides, mmol/L	1.40 (0.95–1.77)	1.31 (0.91–1.92)	1.09 (0.82–1.80)*	1.42 (1.0–2.13)	1.31 (0.93–1.80)
Plasma uric acid, µmol/L	280.79 ± 61.89	306.29 ± 103.76**	336.07 ± 129.00**	303.06 ± 100.01**	296.79 ± 92.94*
Smoking, n (%)	36 (5.8%)	145 (26.7%)**	30 (20.3%)**	52 (23.2%)**	63 (28.0%)**
Alcohol intake, n (%)	47 (7.6%)	120 (22.1%)**	28 (18.9%)**	48 (21.4%)**	44 (19.6%)**
History of hypertension, n (%)	313 (50.8%)	324 (59.7%)*	69 (46.6%)**	138 (61.6%)*	118 (52.4%)
History of diabetes, n (%)	102 (16.6%)	110 (20.3%)	17 (11.5%)	58 (25.9%)*	36 (16.0%)
History of CHD, n (%)	2 (0.3%)	77 (14.2%)**	13 (8.8%)**	38 (17.0%)**	26 (11.6%)**
Telomerase length (Ln-T/S ratio)	1.75 ± 0.71	1.65 ± 0.95	1.62 ± 1.04	1.45 ± 0.95**	1.87 ± 0.87*

Abbreviations: BP, blood pressure; CHD, coronary heart disease. Telomere length is expressed as a relative telomere repeat copy/single-copy gene (T/S) ratio, and Ln(T/S) is natural logarithm transformed leucocyte telomere length T/S ratio. Values are mean ± SD, number (percentage), or median (interquartile range).

*P < 0.05, **P < 0.01, stroke patients *vs*. control subjects. The two-sample *t-*test was used for comparison of continuous variables, the chi-square test for categorical variables, and the Mann–Whitney *U* test for Triglycerides and relative T/S ratio.

Next, the telomere length ratio was divided into the tertile and clinical characteristics by tertile of the telomere length ratio were analyzed in the first stroke study participants (Supplementary Table S1) and 3 subtypes of stroke (Supplementary Table S2), respectively. The results found that the prevalence of conventional vascular risk factors was not significantly different among the highest, middle, and lowest tertile groups, except for blood pressure and fasting glucose.

### Association of leukocyte telomere with risk of first-onset stroke

In the first stroke study, in multivariable analysis adjusting age, gender, and other conventional risk factors, those persons within the lowest tertile of telomere showed a remarkably higher risk for subtype of atherothrombosis (OR 2.33, 95% CI 1.42–3.83; *P* = 0.003), whereas had a lower presence for lacunar infarction (OR 0.49, 95% CI 0.30–0.81; *P* = 0.007) than those within the highest tertile (Table 2). No significant association was found between shorter telomere and risk of hemorrhagic stroke. In secondary analysis, when telomere was treated as a continuous variable, per 1 S.D. decrease of leukocyte telomere length was associated with approximate 48% higher susceptibility to atherothrombotic stroke whereas with 28% decreased susceptibility to lacunar stroke (*P* < 0.05; Table 2).

**Table 2:** Associations between leucocyte telomere length and stroke in the first case/control study of Southern Chinese population.

Variables	Highest tertile (>2.06)	Middle tertile (1.52–2.06)	Lowest tertile (<1.52)	*P* trend	Per 1-S.D. decrease in Ln-transformed telomere length	*P*
**Controls (n = 616)**	206	205	205			
**Total cases (n = 543)**	179	147	217			
ORs (95% CI)						
No adjustment	1.0	0.83 (0.62–1.11)	1.22 (0.92–1.61)	0.03	1.11 (1.00–1.22)	0.048
Model I	1.0	0.79 (0.55–1.13)	1.04 (0.74–1.47)	0.23	1.07 (0.95–1.21)	0.27
Model II	1.0	0.82 (0.56–1.20)	1.05 (0.72–1.51)	0.38	1.07 (0.94–1.22)	0.33
**Hemorrhagic stroke (n = 94)**	33	21	40			
ORs (95% CI)						
No adjustment	1.0	0.64 (0.36–1.14)	1.22 (0.74–2.01)	0.08	1.16 (0.96–1.42)	0.13
Model I	1.0	0.82 (0.34–1.97)	0.91 (0.40–2.07)	0.91	1.08 (0.80–1.46)	0.61
Model II	1.0	0.73 (0.27–1.97)	0.89 (0.36–2.20)	0.82	1.05 (0.75–1.46)	0.78
**Atherothrombotic stroke (n = 224)**	51	67	106			
ORs (95% CI)						
No adjustment	1.0	1.32 (0.87–1.99)	2.09 (1.42–3.07)	0.001	1.39 (1.21–1.60)	<0.001
Model I	1.0	1.47 (0.90–2.40)	2.39 (1.50–3.80)	0.001	1.49 (1.26–1.75)	<0.001
Model II	1.0	1.43 (0.84–2.41)	2.33 (1.42–3.83)	0.003	1.48 (1.24–1.76)	<0.001
**Lacunar infarction (n = 225)**	95	59	71			
ORs (95% CI)						
No adjustment	1.0	0.62 (0.43–0.91)	0.75 (0.52–1.08)	0.04	0.87 (0.75–1.00)	0.053
Model I	1.0	0.50 (0.31–0.79)	0.51 (0.32–0.80)	0.003	0.74 (0.61–0.88)	0.001
Model II	1.0	0.52 (0.31–0.85)	0.49 (0.30–0.81)	0.007	0.72 (0.59–0.88)	0.001

The cut-off values of tertile of leucocyte telomere length (relative T/S ratio) were derived from the control group, with <1.52 for the lowest, 1.52–2.06 for the middle and >2.06 for the highest tertile (as the reference). OR (95% CI) was obtained with multivariate logistic regression analysis.

Model I: adjustment for age, gender, systolic and diastolic BP, fasting glucose, total cholesterol, triglycerides, plasma uric acid, and body mass index.

Model II: adjustment for the covariates mentioned above plus smoking status, alcohol intake, history of hypertension, diabetes, and previous CHD.

In addition, considering that this association between shorter telomere and stroke risk could be biased because of confounding effects from hypertension, coronary heart disease and diabetes, we performed a secondary sensitivity analysis in which persons with hypertension, coronary heart disease, and diabetes were excluded respectively, and the results were not obviously changed comparing to those of the primary analyses (Supplementary Tables S3, S4, and S5).

### Combined effect of telomere attrition and history of hypertension

**Figure 2:**
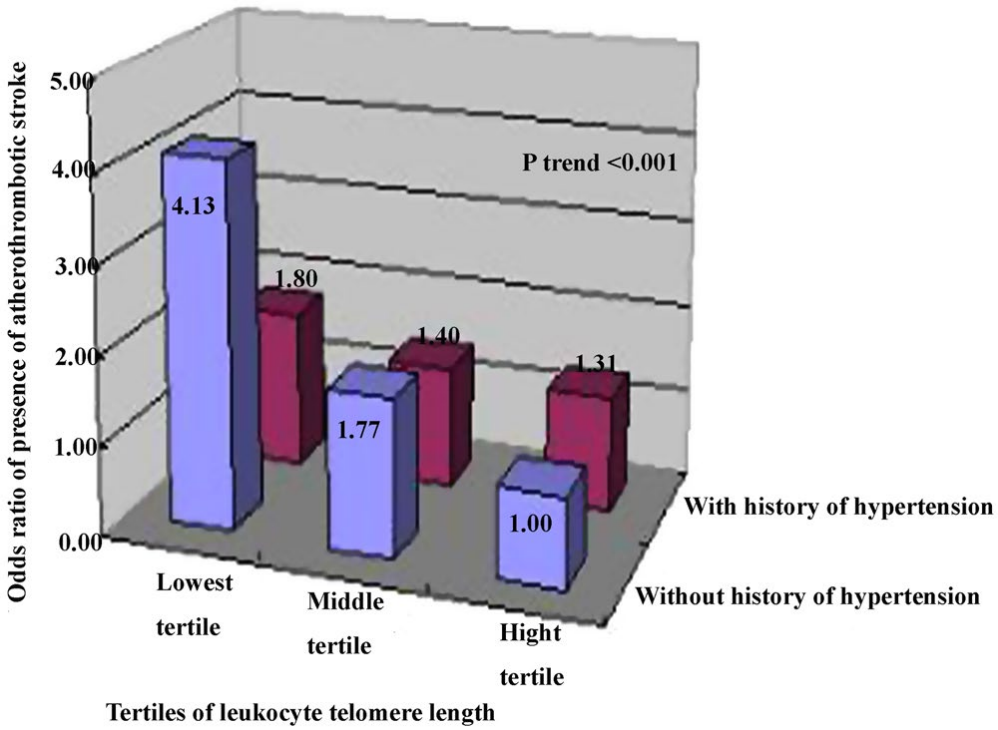
Combined effects of shorter telomere length and hypertension history on stroke risk The cutoff values of tertile of leukocyte telomere length were defined as that in the Table 2. The common reference category for comparisons was subjects at the highest tertile of telomere and no history of hypertension, and ORs (95% CI) were obtained with multivariate logistic regression analysis by adjusting for conventional risk factors as in Table 2.

To explore the modified effects of age, gender, BMI, alcohol intake, smoking, hypertension and diabetes, we conducted deep analyses by evaluating the interaction terms of above variables in the first stroke study (Table 3). As for atherothrombotic stroke, persons had an increased risk in the age groups of 51–60 years (OR 1.59, 95% CI 1.15–2.19) and ≥60 years (OR 1.33, 95% CI 1.10–1.73) for per 1 S.D. decrease of leukocyte telomere length, but not in the younger age group (≤50 years). Associations of stroke risk were positive in both men and women, and in overweight and normal-weight individuals, and stronger in patients without conventional vascular risk factors such as smoking and alcohol intake than those with (Table 3). The tests for interaction of history of hypertension were statistically significant (*P*_interaction_ = 0.001). Next, we examined whether telomere attrition contributed additional information to risk of atherothrombotic stroke for persons without history of hypertension. We observed that persons within the lowest tertile of telomere length and also without hypertension had a 4.13-fold higher risk for atherothrombotic stroke (95% CI 2.01–8.49; *P*_*trend*_ < 0.001) (Fig. 2).

**Table 3:** Stratified association analysis of per 1-S.D. decrease of ln-transformed telomere length in stroke subtypes.

Variables	Atherothrombotic stroke				Lacunar stroke			
	Cases/control	OR (95% CI)	P*	*P*_interaction_^†^	Cases/control	OR (95% CI)	P*	*P*_interaction_^†^
**Gender**								
Male	121/284	1.46 (1.13–1.89)	0.004	0.06	130/284	0.68 (0.52–0.89)	0.005	0.40
Female	103/332	1.43 (1.11–1.85)	0.005		95/332	0.70 (0.52–0.94)	0.019	
**Age**								
≤50	23/99	1.66 (0.88–3.13)	0.12		20/99	0.79 (0.37–1.70)	0.55	0.09
51–60	57/134	1.59 (1.15–2.19)	0.005	0.07	42/134	0.75 (0.50–1.12)	0.16	
≥60	144/383	1.38 (1.10–1.73)	0.006		163/383	0.67 (0.53–0.85)	0.001	
**BMI**								
<25	144/464	1.44 (1.14–1.82)	0.002	0.25	163/464	0.58 (0.45–0.75)	<0.001	0.09
≥25	80/152	1.66 (1.25–2.20)	<0.001		62/152	1.12 (0.80–1.58)	0.51	
**Smoking**								
No	172/580	1.48 (1.22–1.80)	<0.001	0.81	162/580	0.69 (0.55–0.86)	0.001	0.92
Yes	52/36	1.40 (0.91–2.14)	0.12		63/36	0.72 (0.45–1.13)	0.15	
**Alcohol intake**								
No	176/569	1.41 (1.17–1.71)	<0.001	0.79	181/569	0.70 (0.56–0.86)	0.001	0.82
Yes	48/47	1.50 (0.93–2.41)	0.10		44/47	0.74 (0.47–1.16)	0.10	
**Hypertension**								
No	86/303	1.86 (1.42–2.45)	<0.001	0.001	107/303	0.59 (0.43–0.81)	0.001	0.055
Yes	138/313	1.35 (1.06–1.72)	0.016		118/313	0.83 (0.63–1.10)	0.19	
**Diabetes**								
No	166/514	1.40 (1.14–1.71)	0.001	0.55	189/514	0.67 (0.54–0.83)	0.001	0.37
Yes	58/102	1.83 (1.22–2.74)	0.004		36/102	0.97 (0.55–1.72)	0.91	

^*^OR (95% CI) and *P* values were obtained with multivariate logistic regression analysis after adjustment for conventional vascular risk factors, including age, gender, systolic and diastolic BP, fasting glucose, total cholesterol, triglycerides, plasma uric acid, body mass index, smoking status, alcohol intake, history of hypertension, diabetes and previous CHD, except for the stratification variable. ^†^*P*_interaction_ was obtained by evaluating interaction terms.

For lacunar stroke, individuals had a decreased risk in the age groups of ≥60 years (OR 0.67, 95% CI 0.53–0.85) for per 1 S.D. decrease of telomere length, but not in younger age group (<60 years). The association has no gender-specific difference and was only observed in patients without conventional vascular risk factors such as overweight, smoking, alcohol intake, hypertension and diabetes (Table 3). The test for interaction was not significant.

### Association of leukocyte telomere with stroke in the replication stroke study

The associations between telomere and atherothrombotic stroke or lacunar infarction were next replicated in the second independent case-control study population from Southern China (Table 4). The clinical features were summarized in Table S6 (Supplemental materials). Similar as in the first stroke study, the replication analysis showed that after adjusting age, gender, and other conventional risk factors, individuals within the lowest tertile of telomere showed a lower risk for lacunar infarction (OR 0.60, 95% CI 0.39–0.93; *P* = 0.02), and in secondary analysis, per 1 S.D. decrease of leukocyte telomere was associated with approximate 21% decreased susceptibility to lacunar stroke (*P* = 0.003). The multivariable regression analysis also showed a positive association between shorter telomere and the risk for subtype of atherothrombotic stroke (OR 1.52, 95% CI 1.08–2.14; *P* = 0.01). No significant association was found between telomere and hemorrhagic stroke.

**Table 4:** Associations between leucocyte telomere length and stroke in the second case/control study of Southern Chinese population.

Variables^*^	Highest tertile	Middle tertile	Lowest tertile	*P* trend	Per 1-S.D. decrease in Ln-transformed telomere length	*P*
Controls (n = 875)	292	292	291			
Total cases (n = 773)	209	306	258			
ORs (95% CI)	1.00	1.15 (0.86–1.54)	0.85 (0.63–1.15)	0.27	0.92 (0.83–1.03)	0.13
Atherothrombotic stroke (n = 329)	78	130	121			
ORs (95% CI)	1.00	1.38 (0.93–2.04)	1.52 (1.08–2.14)	0.01	1.11 (1.00–1.23)	0.05
Lacunar infarction (n = 222)	71	90	61			
ORs (95% CI)	1.00	1.10 (0.73–1.64)	0.60 (0.39–0.93)	0.02	0.79 (0.68–0.92)	0.003
Hemorrhagic stroke (n = 222)	60	86	76			
ORs (95% CI)	1.00	1.15 (0.72–1.84)	0.84 (0.52–1.36)	0.45	0.97 (0.83–1.14)	0.72

^*^OR (95% CI) was obtained with multivariate logistic regression analysis after adjusting age, gender, BMI, and conventional vascular factors as mentioned in Table 2.

### A meta-analysis of relation between telomere and stroke

The flowchart of the literatures search was shown in Supplementary Figure S1. We retrieved 341 articles from the literature search through June 30, 2017. Of these, 13 articles were identified for full review to assess for their eligibility. The study by Zee *et al*.^10^ was not included because telomere length was not used as a category variable. After detailed assessment, 9 previous studies were included, in which 2 studies reported unclassified stroke events^12,14^, 4 prospective studies reported results on ischemic stroke or its related death^5,9,11,13^, and 3 retrospective studies on ischemic stroke^9,15,16^. Two studies also reported results on hemorrhagic stroke and lacunar stroke^9,15^. Characteristics of these included studies were shown in the Supplementary Table S7. All the original studies were adjusted for multiple covariates including age, gender, BMI, and other risk factors.

**Figure 3:**
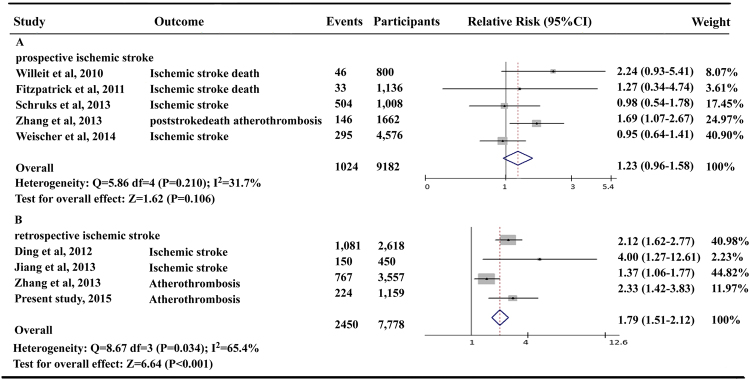
Meta-analysis for the association between shorter telomere length and ischemic stroke by different study design. (**A**) Pooled risk estimate and 95% CI for atherothrombotic stroke susceptibility in prospective cohort studies. (**B**) Pooled risks estimate and 95% CI for atherothrombotic stroke susceptibility in retrospective case-control studies.

Compared with the longest category of leukocyte telomere length level, shorter telomere length was associated with a significant 11% higher risk for stroke (pooled RRs 1.12, 95% CI 1.05–1.19; Supplementary Figure S2), although with significant heterogeneity between studies (I^2^ = 81.1%, *P*_*het*_ < 0.001). A meta-regression analysis was performed to further explore the possible source of heterogeneities in this meta-analysis according to publication year, number of participants, mean age of the study population, gender, ethnicity, study design, country, and medical history of hypertension and diabetic mellitus. Significant between-study heterogeneity was identified mainly from different study design in the original articles (*P* = 0.02), that is, the perspective or retrospective study design. Next, we therefore performed the stratified analysis by the study type of the perspective or retrospective studies. When stratified by study design, significant association between shorter telomere length and ischemic stroke was not found in those prospective studies (pooled RRs 1.03, 95% CI 0.96–1.09) although found in the retrospective studies (pooled RRs 1.81, 95% CI 1.54–2.13) (Fig. 3). There was no evidence of heterogeneity and publication bias in prospective or retrospective studies with Egger’s test (*P* = 0.67 and *P* = 0.36, respectively).

For hemorrhagic stroke, comparing shortest to highest category of telomere length, the pooled relative risk was 1.36 (95% CI 1.04–1.77) without significant heterogeneity (I^2^ = 0.01%, *P*_*het*_ = 0.53) between studies while with a significant publication bias (Egger’s test *P* = 0.06). For lacunar infarction risk, no significant association was found (pooled RRs 1.22, 95% CI 0.99–1.51; I^2^ = 93.6%, *P*_*het*_ < 0.001) (Supplementary Figure S3), along without significant publication bias (*P* = 0.99).

## Discussion

In this stroke study of Southern Chinese population, our data showed a positive association between shorter leukocyte telomere and the risk for atherothrombotic stroke, independent of conventional vascular risk factors such as smoking status, alcohol consumption, hypertension and diabetes history. In particular, this increased risk for atherothrombotic stroke was much pronounced in persons without hypertension, which was up to approximate 4-fold. In contrast, shorter telomere were significantly associated with the lower presence of lacunar infarction and further stratified analysis showed that the effect were mainly present in those old individuals with healthier lifestyle such as no current smoking, no alcohol consumption, and without history of hypertension and diabetes. These findings were also verified in an additional independent stroke case-control study in Southern Chinese population. No significant difference was observed in telomere length between hemorrhagic stroke patients and controls. These findings together imply the complex and key roles of telomere shortening in vascular biology and age-related cerebrovascular diseases.

Our meta-analysis of previous prospective and retrospective studies including our present study showed a 12% increased pooled risk of ischemic stroke and the related death in relation to shorter telomere length, but this association was stronger in the retrospective studies and in the Asian population when further stratified by the study design and ethnicity. The reasons of difference may be partly due to racial and geographical factors, and the incidence of stroke and related vascular risk factors profile in different countries.

Leukocyte telomere length is inheritable and defined at birth^20^. The progression of cellular aging throughout the whole lifetime which can be reflected by the rate of telomere attrition, is related to vascular damage, independently from cardiovascular risk factor exposure^21^. Study by Salpea *et al*.^22^ has reported that a significant difference in leukocyte telomere exists in different geographical areas of Europe. In China, the population structure of ‘North-South’ has been revealed and there is a close relation between the geographical regions and genetic structure of Han ethnicity^23^. The prevalence of stroke is markedly higher in the North (486 per 100,000 person-years) than in the South (136 per 100,000 person-years) because of different lifestyles, genetics, and environments^17,18^. Study by Zhao *et al*. has reported that systolic and diastolic pressures were averagely higher by 7.4 and 6.9 mmHg in the Northern than Southern. Southern individuals had lower BMI, lower intake of sodium and sodium/potassium ratio, while higher intake of vitamins A and C, calcium, and magnesium^24^. Urinary metabolites such as branched-chain amino acids, alanine, lactate, and dimethylglycine, are found to be significantly higher in Northern than Southern Chinese populations and^25^. A small sample study showed that of Chinese population showed that leukocyte telomere length in the Northern people was lower than in the Southern (1.25 ± 0.59 vs. 1.71 ± 0.61)^16^. Similarly, our evidence showed that leukocyte telomere length of individuals resident in Huainan area at the Southern China was 1.75 ± 0.61. Although the geographical difference, in consistent with previous studies from the North of China^9,15^, shorter leukocyte telomere length was correlated with the presence of atherothrombotic stroke no matter in the Southern or Northern Chinese people.

The pathogenesis of atherothrombotic stroke is mainly involved in the formation and rupture of atherosclerotic plaque in large cerebral vessels, such as anterior, middle and posterior cerebral artery, internal and external carotid artery, vertebral and basilar artery, etc. Emerging evidence has shown that telomere attrition, as a marker of biology aging, is linked to endothelial cell apoptosis and senescence and thus contribute to atherogenesis^4^. When atherosclerotic plaque evolves to the late stage, the telomerase functions of vascular smooth muscle cells and endothelial cell change, leading to the progressively telomere shortening, then triggering aging and dysfunction of endothelial cells or smooth muscle cells, which promotes the progression of atherosclerotic plaque^26–29^. However, further experimental and epidemiological evidence needed to assess the causative contribution of telomere attrition to the pathogenesis of atherothrombotic stroke^16^.

Obviously, the pathogenesis of hemorrhagic stroke and lacunar infarction is different from atherothrombotic stroke. Spontaneous (non-traumatic) intracerebral hemorrhage (ICH) is a catastrophic form of stroke with high morbidity and mortality, often resulting from long-term arterial stiffness and arteriolosclerosis, micro-aneurysms, lipohyalinosis, and cerebral amyloid angiopathy occurring in older people^30^. The pathogenic vessels were mostly involved in lenticulostriate arteries which was small deep arteries of the middle cerebral artery, supplying the basal ganglia and deep white matter, and thus resulting in hemorrhagic stroke in deep and lobar brain regions when ruptured. The relationship between telomere and risk of hemorrhagic stroke was not detected in our work and consistently not in our further systematic meta-analysis.

As for lacunar infarction, lacunas result from the occlusion of those small deep penetrating cerebral arteries (with a diameter of <15 mm). Lacunar infarction can be classified as proximal vessel lacunar infarction (branch atheromatous disease) that is usually due to a localized atheromatous lesion at the mouth of the perforating branches of stem arteries or microatheroma in the orifice of the branch, and distal vessel lacunar infarction (small vessel disease) that is primarily due to lipohyalinosis or fibrinoid necrosis of the distal part of small perforating arteries, arterioles, capillaries, and venules^31^. The symptoms of lacunar infarction are usually the least serious in all cerebrovascular diseases whereas the recurrent attacks of lacunar infarction can predict future occurrence of atherothrombotic stroke. Unlike in atherothrombotic stroke, our study found that shorter telomere was associated with lower presence of lacunar infarction and the effect was mainly pronounced in those older individuals with normal BMI and healthier lifestyle such as no current smoking, no alcohol consumption, and without history of hypertension and diabetes. The relationship between telomere and lacunar infarction was also verified in the second independent stroke case-control study in Southern Chinese population. To date, experiments and population data are sparse about telomere and lacunar infarction. Telomere attrition is believed to reflect the accumulation of vascular damage over the lifetime, such as exposure to oxidative and inflammatory stress, at least in part. Neuroimaging examination showed that markers of cerebral small vessel disease are shown in virtually every individual over 60 years of age^32^, however, the evidence from study of the Cerebrovascular Aosta Registry in Northern Italy^33^ found that the prevalence of lacunar infarction increased with aging before 60 years whereas decreased with aging after 69 years; on the other hand, individuals easily suffer from the risk of atherothrombotic stroke with age after 50 years and there was linear correlation between age and risk of atherothrombotic stroke once the age reaches 50 years. Therefore, the complex roles of telomere shortening may be different in the pathogenesis of large and small vessels, which may open a new viewpoint for the treatment of age-related cerebrovascular diseases. But, it is still essential to further verify these findings in larger and prospective stroke cohort.

Lifestyle factors and conventional vascular factors are known to contribute to the development of cardiovascular diseases, and several cross-sectional studies have shown that these risk factors such as smoking, alcohol intake, body weight and physical activity, can also affect the telomere attrition^34,35^. However, Weischer *et al*.^36^ repeatedly measured telomere length with 10 years part in a general populationt (4,576 participants) from the prospective Copenhagen City Heart Study and found that these factors are not associated with telomere length change during 10 years observation. Thus, it still remains inconclusive whether they can modify the relationship between telomere shortening and susceptibility to stroke. The study by Jiang *et al*.^16^ recently reported that in 150 cases with ischemic stroke and 150 healthy siblings (brothers and sisters of stroke patients), shortening telomere is associated with stroke risk but may not be a causal factor. Considering that the sample size was relatively small and a high selected young stroke cases were recruited in Jiang’s study, the generalizability may be cautious. On the other hand, there is suggestive evidence from European Atherosclerosis Research Study II that inherited short telomere in young men is one of potential mechanisms for a paternal history of premature myocardial infarction^19^.

In this study, we showed that telomere length was independently associated with the risk of atherothrombotic stroke and lacunar infarction after adjusting conventional risk factors in a multivariable logistic regression model, including age, body mass index, systolic and diastolic blood pressure, dyslipidemia, diabetes and hypertension. Considering that some complications such as hypertension, diabetes, and coronary heart disease are likely to result in accelerated vascular aging, to further assess the independence of telomere length from these potential effects as a predisposing factor to stroke, secondary sensitivity analysis were further performed in which persons with hypertension, coronary heart disease, and diabetes were excluded respectively. The results were consistent with the primary analyses (supplemental materials). Even so, we cannot draw a causative conclusion although the inherited factor is important. Future studies of stroke in twins would help clarify the underlying pathogenesis of telomere in stroke process.

Some potential limitations in our study must be mentioned. First, since the present study is a case-control design, selection bias of stroke patients and controls cannot be excluded. In the two independent stroke studies in Southern Chinese population, although patients with stroke and age-, gender- and resident area-matched controls were enrolled at the same time and at the same demographic area, some cases and controls were excluded due to incomplete plasma sample, insufficient DNA or poor DNA quality before performing experiment and data analysis. No remarkable differences in clinical characteristics were observed between those excluded and included subjects. However, it would have been more appropriate to match stroke patients and controls according to the age and gender so as to avoid a statistical difference between cases and controls. Second, the sample of stroke patients, particularly the number of hemorrhagic stroke, is small, thus the power is limited to reflect the relation between the telomere length and stroke. Third, in this study we measured mean telomere length of leukocytes which can be affected by changes in leukocyte composition. However, our findings were corroborated by an additional independent case-control stroke study and the meta-analysis of previous studies. Further experimental evidence will be useful for clarifying the role of telomere attrition in vascular risk.

## Clinical Perspectives

Our data provided the first evidence that in Southern Chinese stroke population, shorter leukocyte telomere was independently associated with susceptibility to atherothrombotic stroke, particularly pronounced in those persons without history of hypertension, which is also corroborated by our meta-analysis of previous studies about the association between telomere and stroke. In contrast, shorter telomere length was independently related to a lower presence of lacunar infarction which is further verified in an independent replication case-control stroke study. These findings suggest that telomere attrition may exert a differential role in the pathogenesis of stroke subtypes and serve as a potential genetic marker for the presence of atherothrombotic stroke or lacunar infarction.

## Methods

### Stroke sample population

A case-control study was conducted to assess the relation between leukocyte telomere and stroke. The criteria of diagnosis and recruitment, collection of clinical characteristics, and measurement of plasma biochemical variables were given in details in the Supplemental materials. Initially, a total of 651 consecutive cases with first-onset stroke were recruited from January 2012 to January 2014 from the department of neurology at Huainan First People’s Hospital, Anhui province in Southern China. Stroke was defined by the criteria of World Health Organization (WHO)^37^ and the details were presented in the supplemental materials. The diagnosis of stroke includes neurological examinations and computed tomography (CT) or magnetic resonance imaging (MRI). Three subtypes were recruited: cerebral thrombosis (atherothrombotic), intracerebral hemorrhage (ICH), and lacunar infarction (lacunar).

A total of 651 age-, and gender- and resident area-matched controls were recruited at the same time from January 2012 to January 2014 and at the same demographic area from the local community-based inhabitants. Eligible Individuals were grouped by age with 5-year range, and the controls were randomly chosen from the corresponding matched age group. All recruited controls had no history of neurological condition and used the exclusion criteria as case subjects. Before performing experiment and data analysis, 108 patients and 35 control subjects were excluded because of incomplete plasma data, insufficient DNA or poor DNA quality. No remarkable differences in clinical characteristics were observed between those excluded and included subjects. A total of 543 cases with stroke and 616 controls were included in the present study. The study was approved by Huainan Hospital Ethics Committee and written informed consent was provided. All methods were conducted in accordance with the relevant guidelines and regulations. All participants reported to be Han nationality and their immediate family members within three generations lived in the Huainan area.

An additional independent study comprised 880 consecutive patients with first onset stroke (age 61.6 ± 9.4 years) and 880 age-, gender- and resident region-matched controls (age 61.4 ± 8.0 years) that were enrolled from November 2000 to November 2001 from 3 clinical centers located at Wuhan city and Chongqing city in the Southern China. This study population has been used to investigated risk factors of stroke^38^. The criteria of diagnosis and recruitment and collection of clinical characteristics were same as the first case–control stroke study in Huainan city. After excluding 107 patients and 5 control subjects due to incomplete plasma data, insufficient DNA or poor DNA quality, a total of 773 stroke patients and 875 controls were included in the second replication study. The study was approved by the local ethics committees of collaborating hospitals and all participants were from Han nationality and signed the informed consent.

### Telomere measurement

Relative mean telomere length in circulating leukocyte was calculated as the ratio (T/S) of telomere repeat copy number (T) to single-copy gene copy number β-globin (S) which were measured by a quantitative real-time polymerase chain reaction (qPCR) method^39^. The thermal cycling profiles and primer sequences were described in our previous study^9^. All experiments were conducted by laboratory technician who was blinded to the outcome status.

### Systematic review with a meta-analysis

We performed a meta-analysis of the relation between telomere and stroke risk. A systematic literatures search was conducted using the PubMed, Cochrane library, and EMBASE databases and was further supplemented by manually reviewing the reference of obtained articles up to June 30, 2017. Our systematic review complied with the Meta-analysis of Observational Studies in Epidemiology guidelines (MOOSE)^40^. The details were described in the Supplementary data. In brief, studies were included for meta-analysis based on the following criteria: the exposure was leukocyte telomere length; the outcome was ischemic stroke, or hemorrhagic stroke, or lacunar infarction. The unclassified cerebrovascular disease was excluded. We included case-control studies, cross-sectional studies, nested case-control studies and prospective cohort studies.

Two authors (J.L. and C.F.) independently conducted the data extraction with a standardized form. We used the Newcastle-Ottawa Scale to assess study quality which incorporates characteristics on participant selection, exposure ascertainment, outcome ascertainment, and the potential for confounding, varying from zero to a maximum score of 9.

### Statistical analysis

Data distribution was assessed by the 1-sample Kolmogorov-Smirnov test. The T/S ratio representing leukocyte telomere was transformed by natural logarithm due to a skewed distribution. Values are mean ± SD, medians (interquartile range), or numbers (percentage). Clinical features of cases and control subjects were compared by ANOVA for quantitative variables and χ2 tests for categorical variables. The linear regression model was used to examine the correlation between telomere and age in cases and control subjects.

Telomere length were divided by tertile according to the distribution among control subjects, and the cut-off values were <1.52 for the lowest, 1.52–2.06 for the middle, and >2.06 for the highest tertile. The logistic regression model was used to estimate the association between telomere and stroke by ORs and 95% CI. In multivariate models, we first adjusted for age, gender, BMI, blood pressure, total cholesterol, triglycerides, fasting glucose, plasma uric acid level (model I), and the above variables plus alcohol intake, smoking status, hypertension, coronary heart disease, and diabetes (model II). The significance of multiplicative interaction between telomere length and history of hypertension was assessed by the likelihood-ratio test. Data were analyzed by SPSS Statistics 20.0 (SPSS Inc, Chicago, USA), and *P* < 0.05 was considered to be significant.

In a case-control study, data are usually estimated in odds ratio when the outcome variable of interest is categorical. If *p*_1_ and *p*_2_ respectively are proportion of cases and controls exposed to a risk factor, and then: Odds ${\rm{Ratio}}={\rm{OR}}=\frac{{p}_{1}(1-{p}_{2})}{{p}_{2}(1-{p}_{1})}$, ${\rm{p}}=\frac{{p}_{1}+{p}_{2}}{2}$, the sample size N for estimating an OR is ${\rm{N}}=\frac{{(1+r)}^{2}{({Z}_{\alpha /2}+{Z}_{1-\beta })}^{2}}{r{(lnOR)}^{2}[p(1-p)]}$, in which where *r* is the ratio of case number to control number, *Z*_a/2_ and *Z*_1−β_ are respectively normal parameters for type I error (significance level) and power of study^41^. Our study is interested to estimate the risk of stroke in relation to telomere length; at a significance level of 5% (two-sided), to detect an association with ORs ≥1.30 for stroke risk among individuals within the lowest tertile group of telomere, the first case-control stroke study had >80% power for overall stroke risk, >80% power for hemorrhagic stroke, >86% power for atherothrombotic stroke and >85% power for lacunar infarction, respectively; the replication case-control stroke study had >90% power for overall stroke risk, >84% power for hemorrhagic stroke, >90% power for atherothrombotic stroke, and >88% power for lacunar infarction, respectively.

A meta-analysis was further conducted to assess the relationship between telomere length and stroke with STATA 12.0 software (STATA Corp, College Station, Texas, USA). The multivariate-adjusted hazard ratio and odds ratio presented in the original studies were used to estimate stroke risk. The pooled risk and 95% CI were estimated using random-effect models by comparing the shortest and longest category of leukocyte telomere length. As for the between-study heterogeneity, Der Simonian and Laird’s method was used^42^. The Cochran Q test and the I^2^ statistics were used to examine the heterogeneity across the studies, and I^2^ was calculated with the equation: I^2^ = 100% × (Q − df)/Q. A meta-regression analysis was used to examine the source of heterogeneities and the significance of differences. The Egger’s regression test^43^ and visual inspection of a funnel plot^44^ were conducted to assess the publication bias, which is considered to be significant if the *P* value of Egger’s test is less than 10%.

### Data availability statement

All data generated or analyzed during this study are included in this published article (and its Supplementary Information files).

## Electronic supplementary material

Supplementary materials
