# Supplementary material for: The association of telomere attrition with first-onset stroke in Southern Chinese: a case-control study and meta-analysis
Source: Sci Rep. 2018 Feb 2;8:2290. doi: 10.1038/s41598-018-20434-w (PMC5797248; doi:10.1038/s41598-018-20434-w)
Supplement: Supplementary file 1 — Supplementary materials [file 41598_2018_20434_MOESM1_ESM.doc]

**Supplementary Materials**

**Title:** The association of telomere attrition with first-onset stroke in Southern Chinese: a case-control study and meta-analysis

**Author’s names and affiliations:** Jing Li^1#^, Congrui Feng^2#^, Liang Li^3^, Shujun Yang^4^, Yu Chen^4^, Rutai Hui^4^, Mei Zhang^1*^, Weili Zhang^2, 4*^

**Supplementary methods**

**Diagnosis of stroke**

Stroke was defined according to the World Health Organization (WHO) definition as “rapidly developing clinical signs of focal (or global) disturbance of cerebral function lasting more than 24 hours (unless interrupted by surgery or death) with no apparent cause other than of vascular origin.” [1]. Our study excludes cases of primary cerebral tumor, cerebral metastasis, subdural hematoma, post-seizure palsy, TIA, and brain trauma. The diagnosis of stroke included neurological examination, brain computed tomography (CT) or magnetic resonance imaging (MRI). Three subtypes were recruited: cerebral thrombosis (atherothrombosis), intracerebral hemorrhage (ICH), and lacunar infarction (lacunar). Other types of stroke such as embolic stroke and subarachnoid hemorrhage and systemic diseases such as collagenosis, inflammation, liver, or renal diseases were excluded.

Atherothrombotic subtype of stroke was defined as >50% stenosis or an occlusion of a major brain artery or branch cortical artery confirmed by angiography or duplex sonography and having the clinical features corresponding to cerebral cortical, cerebellar or brain stem dysfunction; embolism without definite cardiac source; infarct locating at the cortical, subcortical, brain stem or cerebellar, with ≥15 mm in diameter on CT or MRI [2]. Lacunar infraction was diagnosed if a patient showed the clinical lacunar syndromes, and a deep focal infraction in brain stem or subcortical region was <15mm in diameter on CT or MRI [3]. Intracerebral hemorrhage (ICH) was diagnosed if a patient had the symptoms of cerebral dysfunction and an area of hyperdensity of the brain parenchyma on CT scan [4].

**Systematic review with a meta-analysis**

We performed a meta-analysis of the relation between telomere length and stroke risk. A systematic literatures search was conducted using the PubMed, Cochrane library, and EMBASE databases and was further supplemented by manually reviewing the reference of obtained articles up to June 30, 2017. The following terms were used: ‘telomere’, ‘telomere length’, ‘stroke’, ‘ischemic stroke’, ‘atherothrombosis’, ‘atherothrombotic stroke’, ‘large-vessel stroke’, ‘small-vessel stroke’, ‘intracranial hemorrhages’, ‘hemorrhagic stroke’ and ‘lacunar infraction’. There is no language restriction for searching and study inclusion.

Studies were included for meta-analysis based on the following criteria: the exposure was leukocyte telomere length; the outcome was ischemic stroke, or hemorrhagic stroke, or lacunar infarction. The unclassified cerebrovascular disease was excluded. We included case-control studies, cross-sectional studies, nested case-control studies and prospective cohort studies.

Two authors (J.L. and C.F.) independently conducted the data extraction with a standardized form. For the included studies, their characteristics were recorded as the following: authors, country of study, publication year, name of study, study design, characteristics of study population (number of participants, number of outcome, age and proportion of men), duration of the follow-up of the prospective studies, median or mean (standard deviation, SD) values for the leukocyte telomere length, reported hazards ratio or odds ratio related to outcome, and adjustment for covariates.

**References**

1. Adams, H.P. Jr. *et al*. Classification of subtype of acute ischemic stroke. Definitions for use in a multicenter clinical trial. TOAST. Trial of Org 10172 in Acute Stroke Treatment. *Stroke.* **24**, 35-41 (1993)
2. Kim, B.J. & Kim, J.S. Ischemic Stroke Subtype Classification: An Asian Viewpoint. *Stroke.* **16,** 8-17 (2014).
3. Fisher, C.M. Lacunes: Small, deep cerebral infarcts. Neurology. **77**, 2104 (2011).
4. Qureshi, A.I., et al. Spontaneous intracerebral hemorrhage. *N Engl J Med*. **344,** 1450–1460 (2001).

**Supplementary tables: 7, online only**

Table S1. Clinical characteristics of all stroke patients by tertile of leukocyte telomere length

Table S2. Clinical characteristics of stroke subtypes by tertile of leukocyte telomere length

Table S3. Association between leukocyte telomere length and stroke in subjects without hypertension

Table S4. Association between leukocyte telomere length and stroke in subjects without diabetes

Table S5. Association between leukocyte telomere length and stroke in subjects without history of CHD

Table S6. Clinical characteristics of the second case/control study in Southern Chinese population

Table S7. Characteristics of studies in the meta-analysis for telomere length and risk of stroke

**Supplementary figures: 3, online only**

Figure S1. The flowchart of the literature searches for studies in the meta-analysis

Figure S2. Meta-analysis of shorter telomere length and stroke risk

Figure S3. Meta-analysis for the association between telomere length and lacunar infarction or hemorrhagic stroke

1. Pooled relative risks (95% CI) of the relationship between telomere length and lacunar infarction;
2. Pooled relative risks (95% CI) of the relationship between telomere length and hemorrhagic stroke.

**Table S1. Clinical characteristics of all** stroke patients by tertile of leukocyte telomere length

|  | Total stroke cases (n=543) | | | |
| --- | --- | --- | --- | --- |
| Characteristics | Highest tertile (>2.06) | Middle tertile (1.52-2.06) | Lowest tertile (<1.52) | *P* value |
| Cases, n | 179 | 147 | 217 |  |
| Age, years | 64.97±12.73 | 66.33±10.95 | 66.38±12.51 | 0.46 |
| Male, n (%) | 99 (55.3%) | 81 (55.1%) | 125 (57.6%) | 0.86 |
| Body mass index, kg/m^2^ | 23.76±2.73 | 23.86±2.84 | 24.05±3.04 | 0.59 |
| Systolic BP, mmHg | 145.57±22.4 | 142.95±21.9 | 149.56±24.5 | 0.04 |
| Diastolic BP, mmHg | 87.63±12.55 | 86.13±13.59 | 90.62±14.70 | 0.01 |
| Glucose, mmol/L | 6.60±3.17 | 6.11±2.22 | 5.91±2.03 | 0.02 |
| Total cholesterol, mmol/L | 4.61±1.43 | 4.40±1.38 | 4.83±2.63 | 0.13 |
| Triglycerides, mmol/L | 1.39 (0.94-1.96) | 1.24 (0.91-1.85) | 1.30 (0.90-1.92) | 0.42 |
| Plasma uric acid, µmol/L | 311.82±112.78 | 309.08±109.89 | 299.83±91.11 | 0.72 |
| Smoking, n (%) | 46 (25.7%) | 34 (23.1%) | 65 (30%) | 0.33 |
| Alcohol intake, n (%) | 36(20.1%) | 27(18.4%) | 57(26.3%) | 0.15 |
| History of hypertension, n (%) | 101(56.4%) | 90(61.2%) | 133(61.3%) | 0.56 |
| History of diabetes, n (%) | 35(19.6%) | 29(19.7%) | 46(21.2%) | 0.91 |
| History of CHD, n (%) | 19(10.6%) | 25(17.0%) | 33(15.2%) | 0.22 |

Abbreviations: BP, blood pressure; CHD, coronary heart disease. Values are mean ± SD, number (percentage), or median (inter-quartile range).

Telomere length is expressed as a relative telomere repeat copy /single-copy gene (T/S) ratio. The cut-off values of tertile of leukocyte telomere length were derived from the control group, and relative T/S ratios were <1.52 for lowest tertile, 1.52-2.06 for the middle tertile and >2.06 for the highest tertile (as the reference).

*P* values were obtained by ANOVA for comparison of continuous variables, the chi-square test for categorical variables, and the Kruskal–Wallis *H* test for Triglycerides.

**Table S2.** Clinical characteristics of stroke subtypes by tertile of leukocyte telomere length

|  | Atherothrombotic stroke (n=224) | | | | Hemorrhagic stroke (n=94) | | | | Lacunar infarction (n=225) | | | |
| --- | --- | --- | --- | --- | --- | --- | --- | --- | --- | --- | --- | --- |
| Characteristics | Highest tertile (>2.06) | Middle tertile (1.52-2.06) | Lowest tertile (<1.52) | *P* | Highest tertile (>2.06) | Middle tertile (1.52-2.06) | Lowest tertile (<1.52) | *P* | Highest tertile (>2.06) | Middle tertile (1.52-2.06) | Lowest tertile (<1.52) | *P* |
| Participants (n) | 51 | 67 | 106 |  | 33 | 21 | 40 |  | 95 | 59 | 71 |  |
| Age (years) | 64.92±12.09 | 66.52±10.84 | 65.91±11.85 | 0.76 | 64.27±15.82 | 61.29±11.39 | 63.87±13.55 | 0.72 | 65.23±11.99 | 67.92±10.56 | 68.51±12.70 | 0.17 |
| Male (n/%) | 28(54.9%) | 33(49.3%) | 60(56.6%) | 0.63 | 16(48.5%) | 12(57.1%) | 26(65.0%) | 0.36 | 55(57.9%) | 36(61.0%) | 39(54.9%) | 0.78 |
| SBP (mmHg) | 144.25±19.07 | 140.99±19.73 | 143.24±18.97 | 0.57 | 159.52±28.09 | 158.43±28.17 | 168.92±27.62 | 0.16 | 141.43±20.10 | 139.66±19.93 | 148.08±24.8 | 0.16 |
| DBP (mmHg) | 85.69±9.61 | 85.51±12.49 | 86.60±11.36 | 0.81 | 95.73±18.12 | 93.48±19.04 | 104.13±16.36 | 0.01 | 85.86±10.46 | 84.22±11.77 | 89.01±13.89 | 0.19 |
| Glucose (mmol/L) | 7.22±2.72 | 6.21±1.99 | 5.94±1.85 | 0.002 | 6.99±3.75 | 6.68±3.21 | 6.75±3.25 | 0.94 | 6.13±3.13 | 5.81±2.04 | 5.39±1.03 | 0.14 |
| Cholesterol (mmol/L) | 4.60±1.52 | 4.22±1.39 | 5.02±3.50 | 0.15 | 4.78±1.31 | 5.01±1.30 | 4.99±1.46 | 0.76 | 4.56±1.42 | 4.39±1.37 | 4.46±1.25 | 0.75 |
| Triglycerides (mmol/L) | 1.48(1.04-2.18) | 1.49(1.03-2.28) | 1.35(0.90-1.98) | 0.45 | 1.06(0.88-1.87) | 1.10(0.74-1.67) | 1.19(0.76-1.89) | 0.89 | 1.42(0.95-2.10) | 1.12(0.85-1.62) | 1.35(0.97-1.88) | 0.07 |
| Uric acid (µmol/L) | 322.35±120.31 | 302.43±105.65 | 294.18±84.11 | 0.48 | 352.12±124.36 | 359.10±177.71 | 312.25±100.25 | 0.48 | 292.17±100.52 | 298.83±75.78 | 301.27±96.24 | 0.86 |
| BMI (kg/m^2^) | 23.98±3.02 | 24.20±3.02 | 24.26±2.94 | 0.86 | 24.26±2.81 | 23.02±3.11 | 23.93±2.90 | 0.31 | 23.47±2.53 | 23.77±2.49 | 23.82±3.29 | 0.67 |
| Smoking (n) | 11(21.6%) | 14(20.9%) | 27(25.5%) | 0.75 | 7(21.2%) | 8(38.1%) | 15(37.5%) | 0.26 | 28(29.5%) | 12(20.3%) | 23(32.4%) | 0.29 |
| Alcohol intake (n) | 10(19.6%) | 12(17.9%) | 26(24.5%) | 0.55 | 8(24.2%) | 5(23.8%) | 15(37.5%) | 0.37 | 18(18.9%) | 10(16.9%) | 16(22.5%) | 0.71 |
| Hypertension (n) | 32(62.7%) | 43(64.2%) | 63(59.4%) | 0.81 | 20(60.6%) | 16(76.5%) | 32(80%) | 0.17 | 49(51.6%) | 31(52.5%) | 38(53.5%) | 0.97 |
| Diabetes (n) | 18(35.3%) | 15(22.4%) | 25(23.6%) | 0.21 | 4(12.1%) | 2(9.5%) | 10(25%) | 0.20 | 13(13.7%) | 12(20.3%) | 11(15.5%) | 0.54 |
| CHD (n) | 8(15.7%) | 13(19.4%) | 17(16.0%) | 0.82 | 4(12.1%) | 3(14.3%) | 6(15%) | 0.94 | 7(7.4%) | 9(15.3%) | 10(14.1%) | 0.24 |

The footnote was the same as the Table S2.

**Table S3. Association between leukocyte telomere length and stroke in subjects without hypertension**

| Variables | Highest tertile  (> 2.06) | Middle tertile  (1.52-2.06) | Lowest tertile  (< 1.52) | *P* for trend |
| --- | --- | --- | --- | --- |
| Control subjects (n=303) | 145 | 84 | 74 |  |
| Total cases (n=219) | 78 | 57 | 84 |  |
| Odds ratio (95% CI) |  |  |  |  |
| Crude model | 1.0 | 1.26 (0.82-1.95) | 2.11 (1.39-3.20) | 0.002 |
| Model I | 1.0 | 0.77 (0.46-1.27) | 1.22 (0.75-2.00) | 0.20 |
| Model II | 1.0 | 0.76 (0.44-1.32) | 1.10 (0.63-1.90) | 0.41 |
| Hemorrhagic stroke (n=26) | 13 | 5 | 8 |  |
| Odds ratio (95% CI) |  |  |  |  |
| Crude model | 1.0 | 0.66 (0.23-1.93) | 1.21 (0.48-3.04) | 0.60 |
| Model I | 1.0 | 0.47 (0.13-1.71) | 0.60 (0.18-2.01) | 0.48 |
| Model II | 1.0 | 0.45 (0.09-2.15) | 0.93 (0.20-4.28) | 0.56 |
| Atherothrombotic stroke (n=86) | 19 | 24 | 43 |  |
| Odds ratio (95% CI) |  |  |  |  |
| Crude model | 1.0 | 2.18 (1.13-4.22) | 4.44 (2.41-8.15) | <0.001 |
| Model I | 1.0 | 1.54 (0.73-3.24) | 3.60 (1.77-7.33) | 0.001 |
| Model II | 1.0 | 1.76 (0.81-3.91) | 4.06 (1.93-8.55) | 0.001 |
| Lacunar infarction (n=107) | 46 | 28 | 33 |  |
| Odds ratio (95% CI) |  |  |  |  |
| Crude model | 1.0 | 1.05 (0.61-1.81) | 1.41 (0.83-2.38) | 0.40 |
| Model I | 1.0 | 0.49 (0.25-0.95) | 0.51 (0.26-1.00) | 0.06 |
| Model II | 1.0 | 0.46 (0.22-0.95) | 0.49 (0.23-1.03) | 0.06 |

The cut-off values of tertile of leucocyte telomere length were derived from the control group, and relative T/S ratios were <1.52 for lowest tertile, 1.52-2.06 for the middle tertile and >2.06 for the highest tertile (as the reference). Odds ratio (95% CI) was obtained with multivariate logistic regression analysis.

Crude Model: no adjustment.

Model I: adjustment for age, gender, systolic and diastolic BP, glucose, total cholesterol, triglycerides, plasma uric acid and BMI.

Model II: adjustment for the covariates mentioned above plus smoking status, alcohol intake, history of diabetes and CHD.

**Table S4. Association between leukocyte telomere length and stroke in subjects without diabetes**

| Variables | Highest tertile  (> 2.06) | Middle tertile  (1.52-2.06) | Lowest tertile  (< 1.52) | *P* for trend |
| --- | --- | --- | --- | --- |
| Control subjects (n=514) | 171 | 173 | 170 |  |
| Total cases (n=433) | 144 | 118 | 171 |  |
| Odds ratio (95% CI) |  |  |  |  |
| Crude model | 1.0 | 0.81 (0.59-1.12) | 1.19 (0.88-1.62) | 0.06 |
| Model I | 1.0 | 0.75 (0.51-1.11) | 0.94 (0.64-1.37) | 0.31 |
| Model II | 1.0 | 0.75 (0.49-1.14) | 0.96 (0.64-1.43) | 0.33 |
| Hemorrhagic stroke (n=78) | 29 | 19 | 30 |  |
| Odds ratio (95% CI) |  |  |  |  |
| Crude model | 1.0 | 0.65 (0.35-1.20) | 1.04 (0.60-1.81) | 0.26 |
| Model I | 1.0 | 0.68 (0.27-1.70) | 0.71 (0.30-1.68) | 0.65 |
| Model II | 1.0 | 0.57 (0.21-1.57) | 0.70 (0.27-1.82) | 0.54 |
| Atherothrombotic stroke (n=166) | 33 | 52 | 81 |  |
| Odds ratio (95% CI) |  |  |  |  |
| Crude model | 1.0 | 1.56 (0.96-2.53) | 2.47 (1.56-3.90) | <0.001 |
| Model I | 1.0 | 1.58 (0.90-2.77) | 2.47 (1.44-4.24) | 0.004 |
| Model II | 1.0 | 1.45 (0.80-2.63) | 2.27 (1.29-3.99) | 0.01 |
| Lacunar infarction (n=189) | 82 | 47 | 60 |  |
| Odds ratio (95% CI) |  |  |  |  |
| Crude model | 1.0 | 0.57 (0.37-0.86) | 0.74 (0.50-1.09) | 0.025 |
| Model I | 1.0 | 0.44 (0.27-0.73) | 0.44 (0.27-0.72) | 0.001 |
| Model II | 1.0 | 0.44 (0.25-0.75) | 0.45 (0.26-0.76) | 0.002 |

The footnote was same as Table S1, except that further adjusting for smoking status, alcohol intake, history of hypertension and CHD in Model II.

**Table S5. Association between leukocyte telomere length and stroke in subjects without history of CHD**

| Variables | Highest tertile  (> 2.06) | Middle tertile  (1.52-2.06) | Lowest tertile  (< 1.52) | *P* for trend |
| --- | --- | --- | --- | --- |
| Control subjects (n=614) | 206 | 203 | 205 |  |
| Total cases (n=466) | 160 | 122 | 389 |  |
| Odds ratio (95% CI) |  |  |  |  |
| Crude model | 1.0 | 0.77 (0.57-1.05) | 1.16 (0.87-1.54) | 0.03 |
| Model I | 1.0 | 0.74 (0.51-1.08) | 0.97 (0.68-1.38) | 0.21 |
| Model II | 1.0 | 0.57 (0.84-1.23) | 1.04 (0.72-1.51) | 0.49 |
| Hemorrhagic stroke (n=81) | 29 | 18 | 34 |  |
| Odds ratio (95% CI) |  |  |  |  |
| Crude model | 1.0 | 0.63 (0.34-1.17) | 1.18 (0.69-2.00) | 0.12 |
| Model I | 1.0 | 0.78 (0.31-2.00) | 0.89 (0.37-2.14) | 0.87 |
| Model II | 1.0 | 0.77 (0.29-2.10) | 0.87 (0.34-2.25) | 0.88 |
| Atherothrombotic stroke (n=186) | 43 | 54 | 89 |  |
| Odds ratio (95% CI) |  |  |  |  |
| Crude model | 1.0 | 1.27 (0.82-1.99) | 2.08 (1.38-3.14) | <0.001 |
| Model I | 1.0 | 1.38 (0.82-2.33) | 2.31 (1.41-3.78) | 0.002 |
| Model II | 1.0 | 1.50 (0.88-2.54) | 2.33 (1.41-3.85) | 0.003 |
| Lacunar infarction (n=199) | 88 | 50 | 61 |  |
| Odds ratio (95% CI) |  |  |  |  |
| Crude model | 1.0 | 0.58 (0.39-0.86) | 0.70 (0.48-1.02) | 0.018 |
| Model I | 1.0 | 0.46 (0.28-0.75) | 0.46 (0.29-0.75) | 0.001 |
| Model II | 1.0 | 0.54 (0.33-0.89) | 0.49 (0.30-0.80) | 0.009 |

The footnote was same as Table S1, except that further adjusting for smoking status, alcohol intake, history of hypertension and diabetes in Model II.

**Table S6. Clinical characteristics of the second case/control study in Southern Chinese population**

| Characteristics | Control subjects (n=875) | Stroke patients | | | |
| --- | --- | --- | --- | --- | --- |
|  |  | Total cases  (n=773) | Hemorrhagic stroke (n=222) | Atherothrombotic stroke (n=329) | Lacunar infarction (n=222) |
| Age, years | 61.4 ± 8.0 | 61.6 ± 9.4 | 58.8 ± 9.9** | 62.7 ± 9.5* | 62.5 ± 8.1 |
| Male, n (%) | 465 (63.1%) | 489 (65.3%)* | 146 (65.8%)* | 211 (64.1%)* | 132 (65.5%)* |
| Body mass index, kg/m^2^ | 23.76 ± 3.07 | 23.99 ± 3.44 | 23.93 ± 3.51 | 23.89 ± 3.56 | 24.20 ± 3.16 |
| Systolic BP, mmHg | 131 ± 17 | 146 ± 23** | 150 ± 25** | 146 ± 22** | 142 ± 20** |
| Diastolic BP, mmHg | 79 ± 10 | 87 ± 13** | 91 ±14** | 86 ± 12** | 84 ± 11** |
| Glucose, mmol/L | 5.66 ± 1.54 | 6.28 ± 2.38** | 6.44 ± 2.38** | 6.26 ± 2.30** | 6.15 ± 2.51** |
| Total cholesterol, mmol/L | 4.96 ± 1.01 | 4.68 ± 0.99** | 4.43 ± 0.97** | 4.74 ± 0.93** | 4.85 ± 1.04 |
| Triglycerides, mmol/L | 1.42 (1.02-2.06) | 1.63 (1.19-2.31) | 1.45 (1.13-2.04)** | 1.72 (1.21-2.37) | 1.71 (1.23-2.53)** |
| Plasma uric acid, µmol/L | 274.95 ± 70.72 | 276.18 ± 83.15 | 256.75 ± 84.71** | 281.09 ± 80.91 | 288.33 ± 81.82* |
| Smoking, n (%) | 157 (17.9%) | 169 (21.9%)* | 58 (26.1%)** | 64 (19.5%) | 47 (21.2%) |
| Alcohol intake, n (%) | 170 (19.4%) | 142 (18.4%) | 55 (24.8%) | 50 (15.2%) | 37 (16.7%) |
| History of hypertension, n (%) | 171 (19.5%) | 504 (65.2%)** | 148 (66.7%)** | 217 (66.0%)** | 139 (62.6%)** |
| History of diabetes, n (%) | 70 (8.0%) | 142 (18.4%)** | 46 (20.7%)** | 61 (18.5%)** | 35 (15.8%)** |
| History of CHD, n (%) | 47 (5.4%) | 109 (14.1%)** | 21 (9.5%)* | 64 (19.5%)** | 24 (10.8%)** |

Abbreviations: BP, blood pressure; CHD, coronary heart disease. Values are mean ± SD, number (percentage), or median (interquartile range).

**P*<0.05, ***P*<0.01, stroke patients *vs.* control subjects. The two-sample *t-*test was used for comparison of continuous variables, the chi-square test for categorical variables, and the Mann–Whitney *U* test for Triglycerides.

**Table S7. Characteristics of studies in the meta-analysis for** telomere length and risk of stroke

| **Source** | **Country** | **Study design** | **Follow-up**  **(years)** | **Outcome,**  **No.** | **Participants,**  **No.** | **Age, years**  **Mean (SD)** | **Men (%)** | | **Assay**  **method** | **Mean telomere length (SD)** | **Quality score** |
| --- | --- | --- | --- | --- | --- | --- | --- | --- | --- | --- | --- |
| **Prospective studies** | | | | | | | |  | | | |
| Willeit et al. 2010 | Italy | Prospective population based Bruneck Study | 10 | Ischemic stroke death: 46 | 800 | 62.7 (11.1) | 49 | | qPCR | 1.48 ± 0.8 | 9 |
| Fitzpatrick et al. 2011 | United States | The Cardiovascular Health Study | 6.1 | Ischemic stroke death: 33 | 1136 | 73.9 (4.7) | 39.5 | | SB | 6.3 ± 0.6 | 8 |
| Fyhrquist et al. 2011 | Finnish | the LIFE study | 4.0 | Stroke: 43 | 1271 | 55-80 | 47.4 | | qPCR | Women:8.1(0.81)Men:8.2 (0.80) | 7 |
| Schurks et al. 2013 | United States | Nested case-control study in the Nurses’ Health Study | 16 | Ischemic stroke: 504 | 1008 | 61.4 (5.9) | 0 | | qPCR | Median: 0.540 | 8 |
| Zhang et al. 2013 | China | the Multicenter Chinese Stroke Study | 4.5 | Atherothrombotic stroke death: 146 | 1662 | 60.3 (9.2) | 63.4 | | qPCR | case: 0.44; control: 0.53 | 6 |
| Weischer et al. 2014 | United States | Population based the general population | 10 | Ischemic stroke: 295 | 4576 | 47-76 | 42.5 | | qPCR | -1.59-1.38 | 8 |
| **Retrospective studies** | | | | | | | |  | | | |
| Fitzpatrick et al. 2007 | United states | Case-control study | - | Stroke: 42 | 419 | 74.2 (5.2) | 41.1 | | SB | 6.3±0.62 | 8 |
| Ding et al. 2012 | China | Case-control study | - | Overall stroke:1309  ischemic stroke: 1081  thrombosis: 686  lacunar: 395  hemorrhagic stroke: 228 | 2618 | 61.4 (9.8) | 66 | | qPCR | Median: case: 1.25  control: 1.41 | 7 |
|  |  |  |  |  |  |  |  | |  |  |  |
| Continued Table S7 |  |  |  |  |  |  |  | |  |  |  |
| **Source** | **Country** | **Study design** | **Follow-up**  **(years)** | **Outcome,**  **No.** | **Participants,**  **No.** | **Age, years**  **Mean (SD)** | **Men (%)** | | **Assay**  **method** | **Mean telomere length (SD)** | **Quality score** |
| Jiang et al. 2013 | China | Case-control study | - | Ischemic stroke: 150 | 450 | 52.2 (8.1) | 74 | | qPCR | 1.32 | 6 |
| Zhang et al. 2013 | China | Case-control study | - | Atherothrombosis: 767  Lacunar infarction: 503  Hemorrhagic stroke: 486 | 3557 | 59.7 (8.2) | 57 | | qPCR | case: 0.44  control: 0.53 | 6 |

Abbreviation: LIFE, Lifestyle Interventions and Independence for Elders; qPCR, quantitative polymerase chain reaction; SB, southern blotting; Quality score based Newcastle-Ottawa scale.


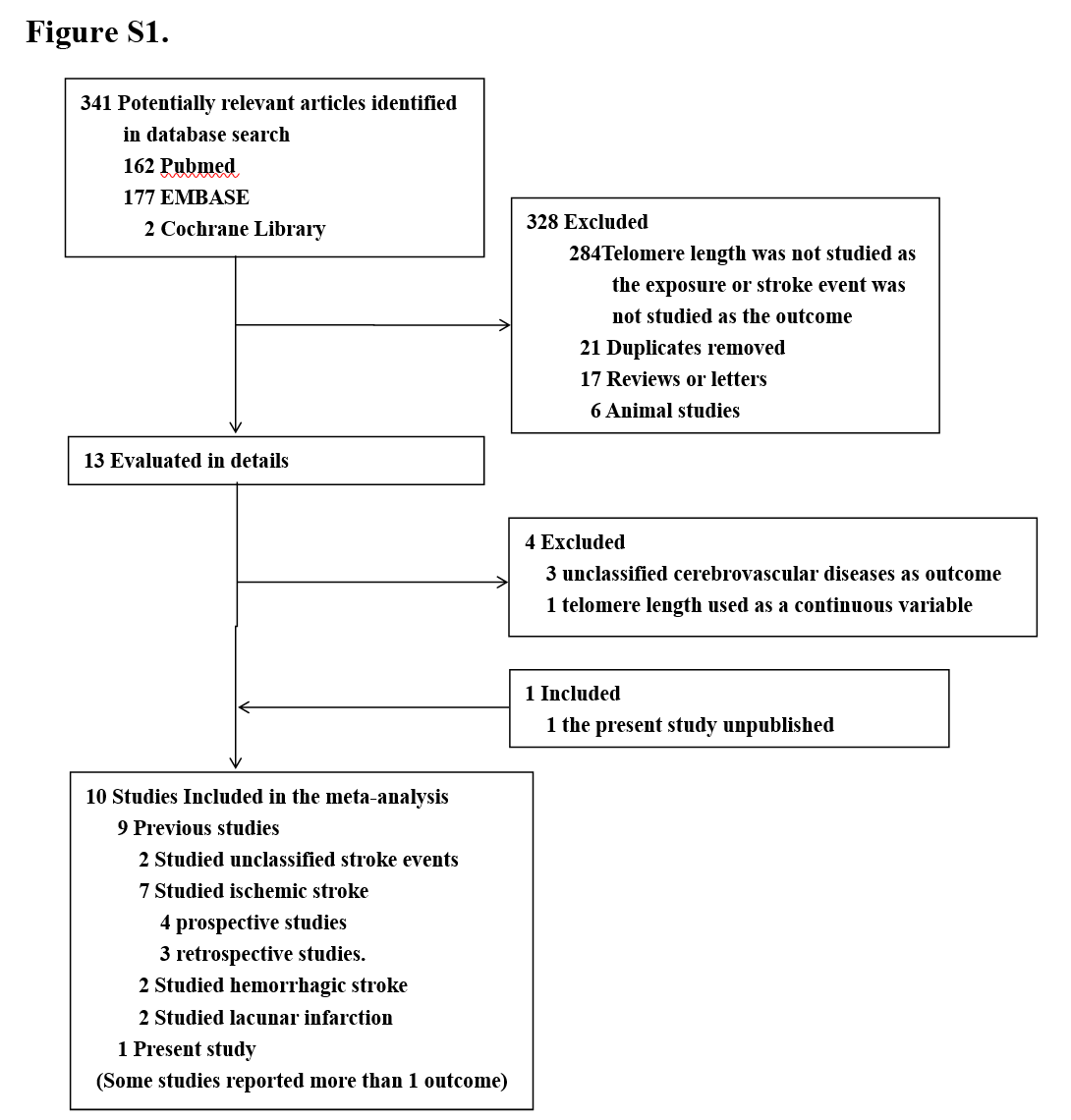


**Figure S1. Flowchart of selection of studies for meta-analysis.**

Literature search was conducted to identify articles up to 30 June, 2017.

**
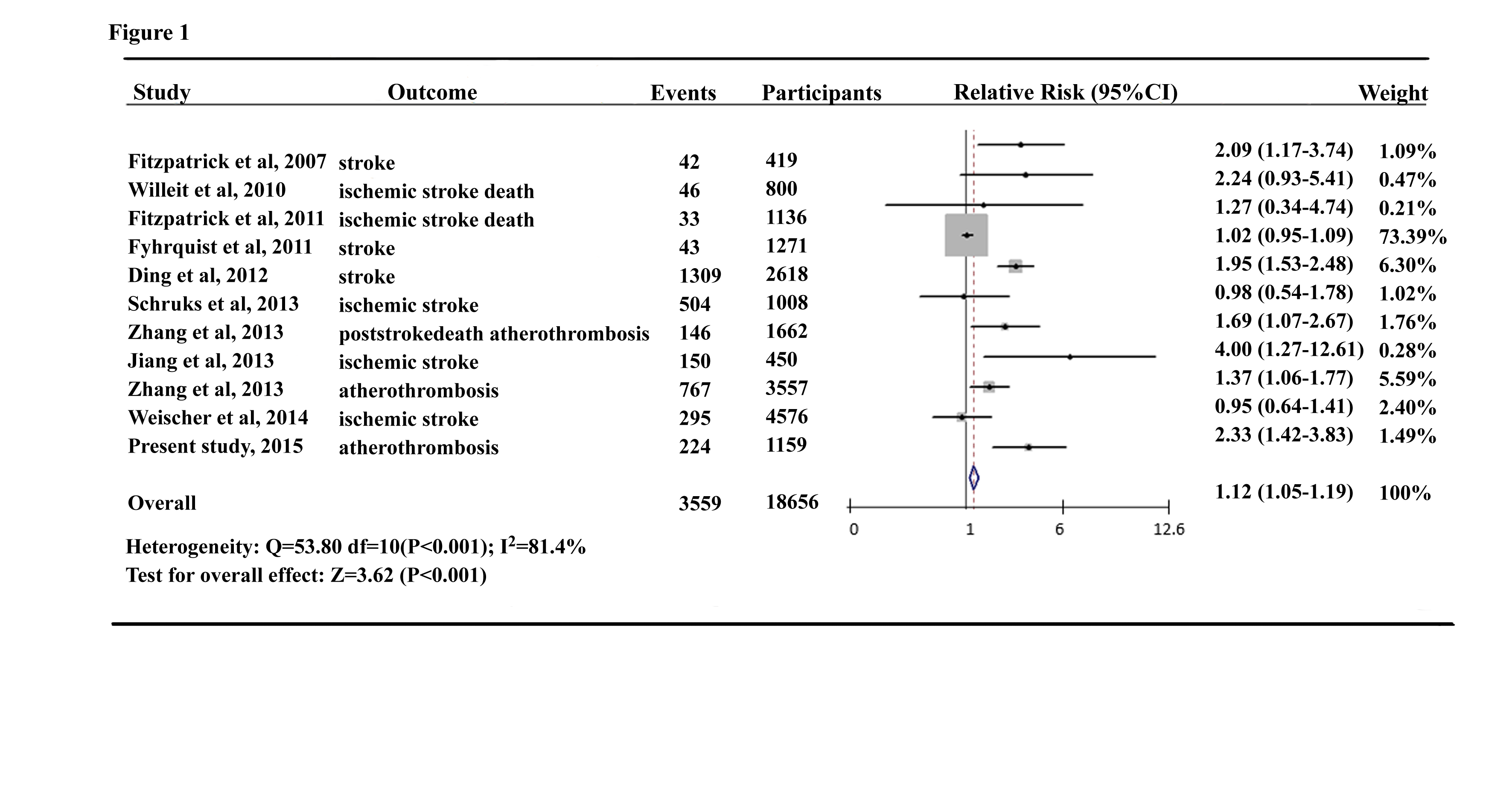
**

**Figure S2. Meta-analysis of shorter telomere length and stroke risk**

**
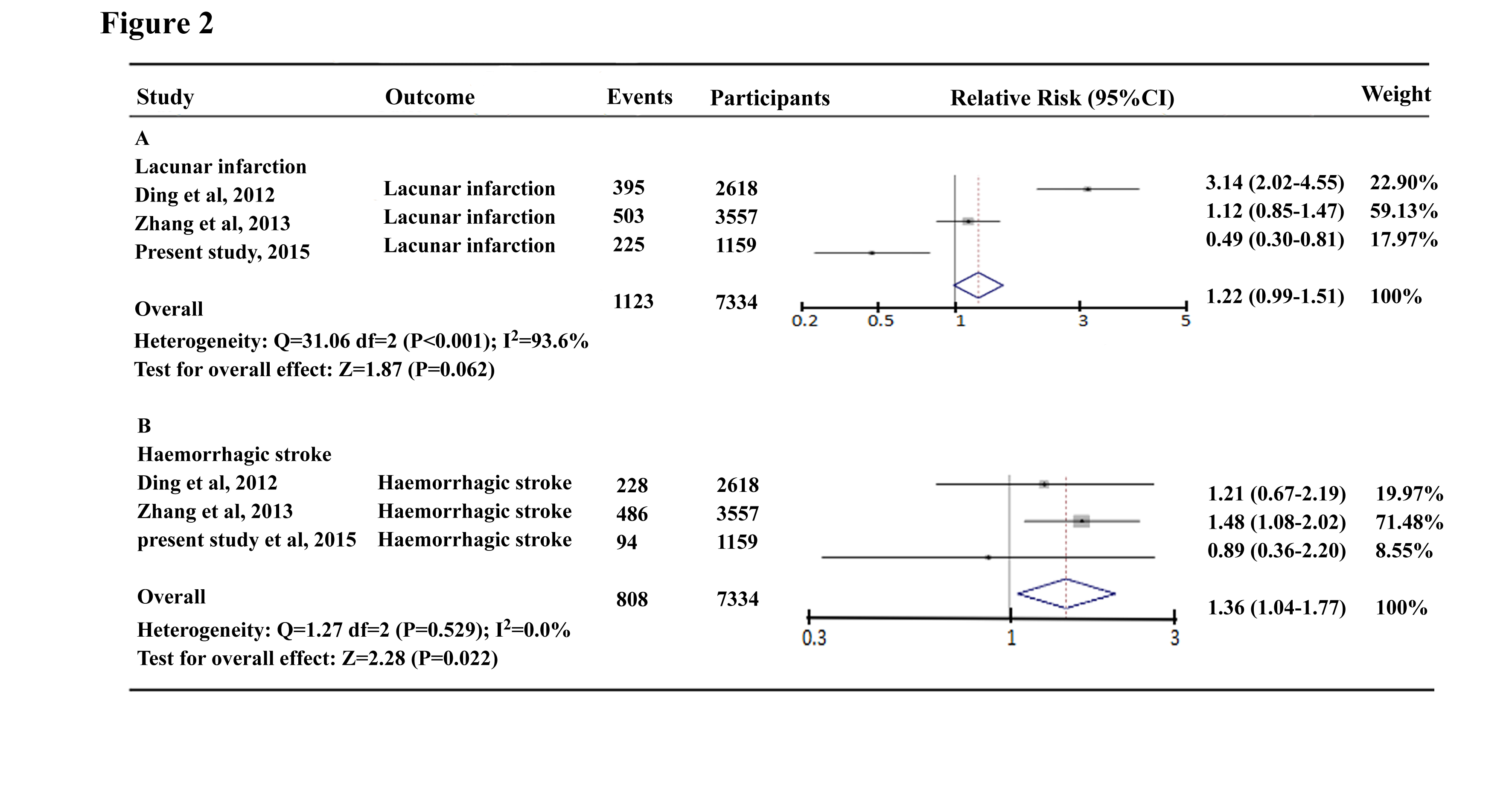
**

**Figure S3. Meta-analysis for the association between telomere length and lacunar infarction or hemorrhagic stroke**

1. Pooled relative risks (95% CI) of the relationship between telomere length and lacunar infarction;
2. Pooled relative risks (95% CI) of the relationship between telomere length and hemorrhagic stroke.
